# Iron-Doped (La,Sr)MnO_3_ Manganites as Promising Mediators of Self-Controlled Magnetic Nanohyperthermia

**DOI:** 10.1186/s11671-015-1223-6

**Published:** 2016-01-14

**Authors:** Yulia Shlapa, Mykola Kulyk, Viktor Kalita, Taras Polek, Alexandr Tovstolytkin, Jean-Marc Greneche, Sergii Solopan, Anatolii Belous

**Affiliations:** V. I. Vernadskii Institute of General and Inorganic Chemistry of the NAS of Ukraine, 32/34 Palladina Ave., Kyiv, 03142 Ukraine; Institute of Physics of the NAS of Ukraine, 46 Nauky Ave., Kyiv, 03028 Ukraine; Institute of Magnetism of the NAS of Ukraine and MES of Ukraine, 36-b Vernadsky Ave., Kyiv, 03142 Ukraine; LUNAM, Institut des Molécules et Matériaux du Mans (IMMM UMR CNRS 6283), Université du Maine, 72085 Le Mans, Cedex France

**Keywords:** Magnetic nanohyperthermia, Manganite nanoparticles, Crystalline structure, Mössbauer spectroscopy, Curie temperature, Specific loss power, 61.46.Df, 75.75.Cd, 81.07. Bc

## Abstract

Fe-doped La_0.77_Sr_0.23_Mn_1 *− y*_Fe_*y*_O_3_ nanoparticles have been synthesized by sol-gel method, and ceramic samples based on them were sintered at 1613 K. Crystallographic and magnetic properties of obtained nanoparticles and ceramic samples have been studied. It has been established that cell volume for nanoparticles increases with growing of iron content, while this dependence displays an opposite trend in the case of ceramic samples. Mössbauer investigations have shown that in all samples, the oxidation state of iron is +3. According to magnetic studies, at room temperature, both nanoparticles and ceramic samples with *y* ≤ 0.06 display superparamagnetic properties and samples with *y* ≥ 0.08 are paramagnetic. Magnetic fluids based on La_0.77_Sr_0.23_Mn_1 *− y*_Fe_*y*_O_3_ nanoparticles and aqua solution of agarose have been prepared. It has been established that heating efficiency of nanoparticles under an alternating magnetic field decreases with growing of iron content.

## Background

Nanoparticles and composite materials based on them have unique electrical, chemical, and magnetic properties. Many scientific papers have stressed high potential of their application in different fields of science and technology [1–3]. One of the most promising directions of magnetic nanoparticle investigations is the opportunity to use them in engineering, medicine, and biology, particularly for creation of new magnetic recording systems, for biological fluid purification, for drug and gene delivery, and for hyperthermia [4–6]. Magnetic hyperthermia is a treatment of oncology tumors by injecting magnetic nanoparticles into them and subsequent local heating of the areas of high concentrations of nanoparticles under the influence of an external alternating magnetic field [7]. To be applicable as mediators of nanohyperthermia, the nanoparticles have to satisfy a number of requirements: they have to be single domain, weakly agglomerated, and small in size and display superparamagnetic properties [6]. In addition, they have to demonstrate high heating efficiency under an alternating magnetic field to be able to heat the tumor area to 42–45 °C (optimal for destroying the tumors) [3, 8].

It is known that magnetite nanoparticles (Fe_3_O_4_) with spinel structure have already found some practical application in medicine [9]. However, Mössbauer investigations show that Fe_3_O_4_ nanoparticles are non-stable: Fe^2+^ partially oxidizes to Fe^3+^, and this leads to creation of maghemite (γ-Fe_2_O_3_) phase [10]. One more essential drawback of magnetite is the fact that the transition temperature from magnetically ordered to non-magnetic state (Curie temperature) is quite high: *T*_*C*_ = 585 °C [11]. Since magnetic-field-induced heating is only operative in magnetically ordered state [3], high Curie temperature may give rise to uncontrolled and non-uniform heating of tumors to high temperatures, which, in turn, may lead to destroying the healthy tissues.

The issue of non-uniform heating can be solved by using materials with Curie temperature in the range of 42–45 °C as the mediators of hyperthermia. In this case, the magnetic nanoparticles are expected to heat up under the influence of an alternating magnetic field only to this transition temperature and, thus, the risk of tissue overheating will be strongly reduced [3]. From this point of view, the promising materials are lanthanum-strontium manganites La_1 − *x*_Sr_*x*_MnO_3_, which crystallize in perovskite structure. Their Curie temperature depends on the chemical composition and can be adjusted by partial substitution of La by Sr to meet the requirements needed for hyperthermia [3, 8].

LaMnO_3_ manganite crystallizes in the distorted perovskite structure and displays antiferromagnetic properties. A partial substitution of lanthanum by strontium gives rise to a conversion of a part of Mn^3+^ ions into Mn^4+^ ones. Since these manganese cations occupy equal positions in crystalline lattice, an electron transfer occurs through 2*p* orbitals of O^2−^ anion from Mn^3+^ cations to Mn^4+^ cations and vice versa. Due to this double-exchange mechanism, La_1 − *x*_Sr_*x*_MnO_3_-substituted manganites acquire ferromagnetic properties [12].

Sufficiently small manganite nanoparticles, which are single domain, can display superparamagnetic properties [13]. Under the influence of temperature, the magnetic moment of such particles changes its direction in a random way, which makes the resulting magnetic moment to be zero in the absence of an external magnetic field. It allows the prevention of magnetic interaction between individual nanoparticles, thus decreasing their agglomeration.

It should be noted that the substitutions of lanthanum by strontium usually result in quite strong changes of the Curie temperature of these compounds. At the same time, partial substitution of Mn^3+^ ions by Fe^3+^ ones, where the ionic radius of the latter (0.645 Å) is close to that of the former (0.65 Å) [14], allows fine tuning of the Curie point and makes it possible to obtain the *T*_*C*_ values necessary for hyperthermia.

It is known that synthesis method and heat treatment conditions can substantially influence the magnetic parameters of substituted manganites [15–17]. To date, the researchers’ efforts have mainly been directed towards the studies of manganites synthesized by solid-phase method. A serious drawback of this method is that it passes through the formation of a large number of intermediate phases [18, 19]. In contrast to the solid-phase method, sol-gel technique is based on the use of a homogeneous mixture of reagents, occurs without formation of intermediate phases, and allows obtaining single-phase product with small particle size and narrow size distribution [20–24].

The aim of this work is the synthesis of the samples of La_0.77_Sr_0.23_Mn_1 *− y*_Fe_*y*_O_3_ (*y* = 0–0.1) manganites by sol-gel method, investigation of their structural and magnetic properties, and elucidation of the regularities of the transformation of structural and magnetic properties with changing the Fe content.

## Methods

Iron-doped manganite nanoparticles were synthesized by sol-gel method. Water-soluble salts La(NO_3_)_3_, Sr(NO_3_)_3_, Mn(NO_3_)_2_, and Fe(NO_3_)_3_ were used as starting reagents. Necessary molar amounts of raw reagents were dissolved in bidistilled water. Citric acid and ethylene glycol were added as gel-forming additives. Obtained reaction mixture was heated with stirring at 80 °C. As a result, polyesterification reaction took place and polymer gel was formed. An amorphous precursor (La,Sr)(Mn,Fe)O_3_ was created as a result of pyrolysis of this gel at 200 °C. It was subjected to further heat treatment at 800 °C for 2 h.

Along with La_0.77_Sr_0.23_Mn_1 *− y*_Fe_*y*_O_3_ nanoparticles, ceramic samples based on them were studied in the work. To obtain the ceramics, the manganite nanoparticles were pressed into the tablets (7 × 7 mm) with the addition of 5 % polyvinyl alcohol aqua solution. These tablets were sintered in the air at 1613 K for 2 h.

X-ray diffraction (XRD) studies were carried out using a DRON-4 diffractometer (CuKα radiation). Crystallographic parameters were calculated by Rietveld method using FULL-PROF software package.

Particle size and morphology were studied by transmission electron microscope (TEM) JEOL JEM-1400. Particle size distribution was obtained by means of analysis of TEM images using Image Tool 3 and OriginPro 8.5 SR1 software packages. Mathematic modeling techniques described in [25] were used for calculating particle size distribution.

The synthesized nanoparticles were investigated by ^57^Fe Mössbauer spectrometry: the spectra were recorded at 300 K in a transmission geometry using ^57^Co/Rh γ-ray source mounted on an electromagnetic drive with a triangular velocity form. The samples consisted of a thin powdered layer containing 5 mg Fe/cm^2^. The obtained spectra were analyzed by a least square fitting method using Lorentzian function.

Magnetic measurements were performed in the 120–370 K temperature range using a LDJ-9500 vibrating sample magnetometer. For the calorimetric determination of specific loss power which is released on the exposure of an ensemble of the particles to alternating magnetic field, the ferrofluids based on synthesized magnetic nanoparticles (50 mg/mL) were prepared using 0.1 % aqueous agarose solutions.

To investigate the heating time vs temperature dependences, the obtained magnetic fluids were placed into the magnetic coil, which provided an alternating magnetic field with a frequency of 400 kHz and an amplitude of up to 9.5 kA/m. All measurements were carried out according to the procedure described in [26]. Specific loss power (SLP) values were calculated by the formula:

$$ \mathrm{S}\mathrm{L}\mathrm{P}=\frac{C_{\mathrm{Fluid}}\cdot {V}_S}{m_{\mathrm{powder}}}\cdot \frac{\mathrm{d}T}{\mathrm{d}\tau } $$

where d*T*/d*τ* is the initial slope of the temperature vs time dependence, *C*_Fluid_ and *V*_*s*_ are the volumetric specific heat and volume of the solution, respectively, and *m*_powder_ is the mass of magnetic material in the fluid.

## Results and Discussion

**Fig. 1:**
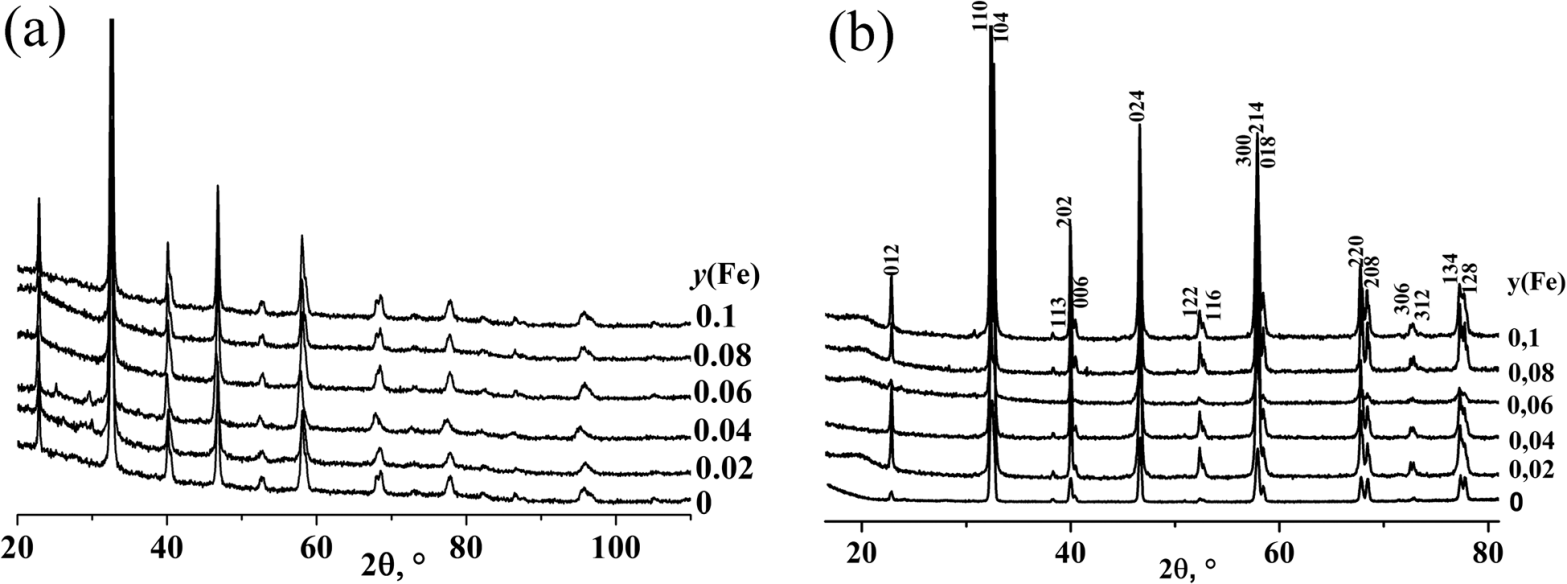
XRD patterns for La_0.77_Sr_0.23_Mn_1 *− y*_Fe_*y*_O_3_ nanoparticles (**a**) and ceramic samples based on them (**b**)

**Fig. 2:**
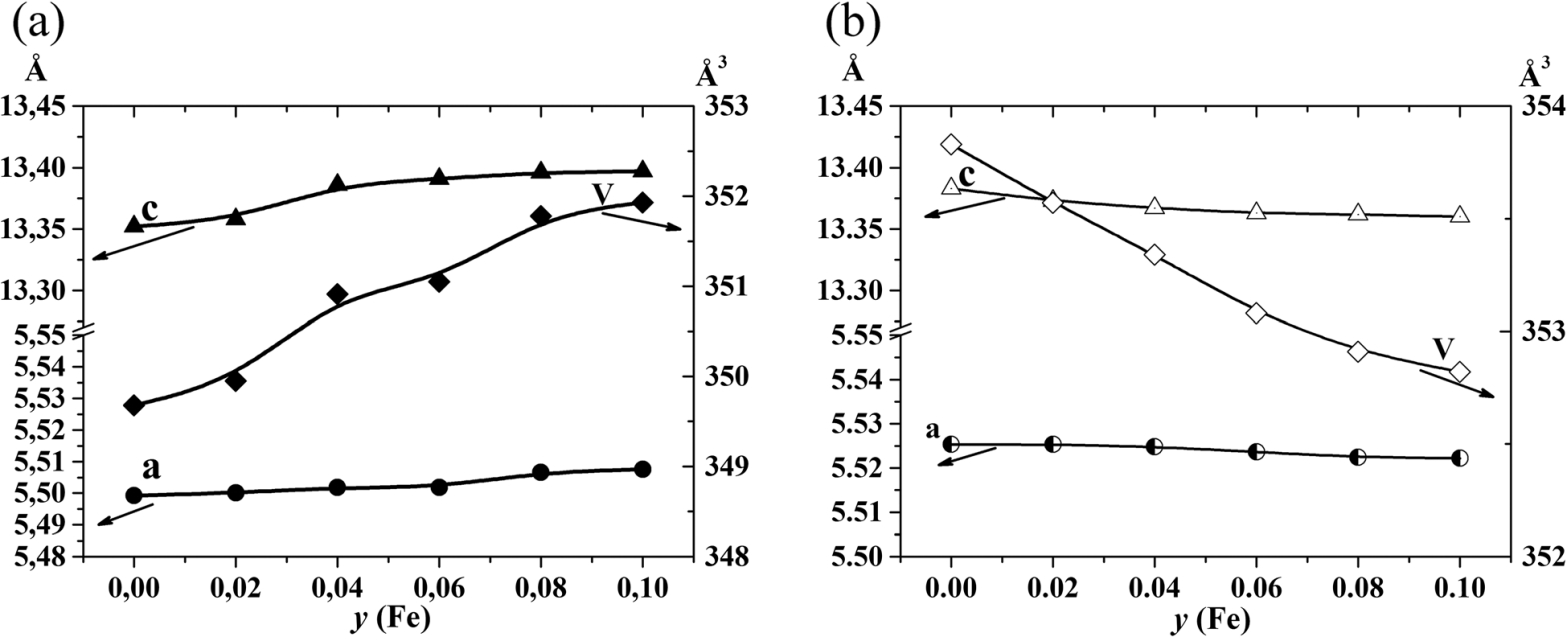
The unit cell parameters vs iron content dependences for La_0.77_Sr_0.23_Mn_1 *− y*_Fe_*y*_O_3_ nanoparticles (**a**) and ceramic samples based on them (**b**)

XRD studies were carried out for La_0.77_Sr_0.23_Mn_1 *− y*_Fe_*y*_O_3_ (*y* = 0–0.1) manganite nanoparticles and ceramic samples based on them. The results of the studies are shown in Fig. 1a, b, respectively. According to XRD investigations, all samples are single phase and crystallize in the distorted perovskite structure. Crystallographic parameters for all the samples were calculated by Rietveld method, and obtained results were summarized in Table 1. As seen from the table, the unit cell volume of the nanoparticles increases with growing of iron content. Taking into account that the Mn^3+^ and Mn^4+^ ionic radii are 0.65 and 0.53 Å, respectively [14], and the ionic radius of Fe^3+^ ions is 0.645 Å, one can conclude that in this case, the heterovalent substitution of smaller Mn^4+^ ions by larger Fe^3+^ ions takes place in accordance with the scheme La_0.77_^3+^Sr_0.23_^2+^Mn_0.77_^3+^Mn_0.23 − *y*_^4+^Fe_*y*_^3+^O_3 − *y*/2_. A reverse dependence is observed for the ceramic samples (Fig. 2). Namely, isovalent substitution of larger Mn^3+^ ions by Fe^3+^ ions is believed to occur according to the scheme La_0.77_^3+^Sr_0.23_^2+^Mn_0.77 − *y*_^3+^Mn_0.23_^4+^Fe_*y*_^3+^O_3_.

**Table 1:** Crystallographic parameters of La_0.77_Sr_0.23_Mn_1 − *y*_Fe_*y*_O_3_ (*y* = 0–0.1) nanoparticles and ceramic samples based on them

	0	0.02	0.04	0.06	0.08	0.10
Nanoparticles						
a. Å	5.4992(2)	5.5001(8)	5.5018 (8)	5.5019(7)	5.5066(9)	5.5076(7)
c. Å	13.352(1)	13.358(2)	13.386(3)	13.391(3)	13.396(3)	13.397(2)
V. Å^3^	349.68(4)	349.95(9)	350.91(8)	351.05(8)	351.78(1)	351.93(8)
R_B_. %	6.1	6.42	5.32	5.81	7.51	5.62
R_f_. %	7.83	8.46	6.65	7.45	9.64	7.39
Ceramic samples						
a. Å	5.5253(2)	5.5253(1)	5.5248(1)	5.5236(2)	5.5224(1)	5.5222(2)
c. Å	13.383(6)	13.373(1)	13.367(3)	13.363(4)	13.362(4)	13.360(2)
V. Å^3^	353.83(2)	353.57(2)	353.34(1)	353.08(8)	352.91(1)	352.82(8)
R_B_. %	5.6	3.8	4.2	2.7	5.37	4.57
R_f_. %	6.9	4.9	5.4	4.92	9.73	8.76

**Fig. 3:**
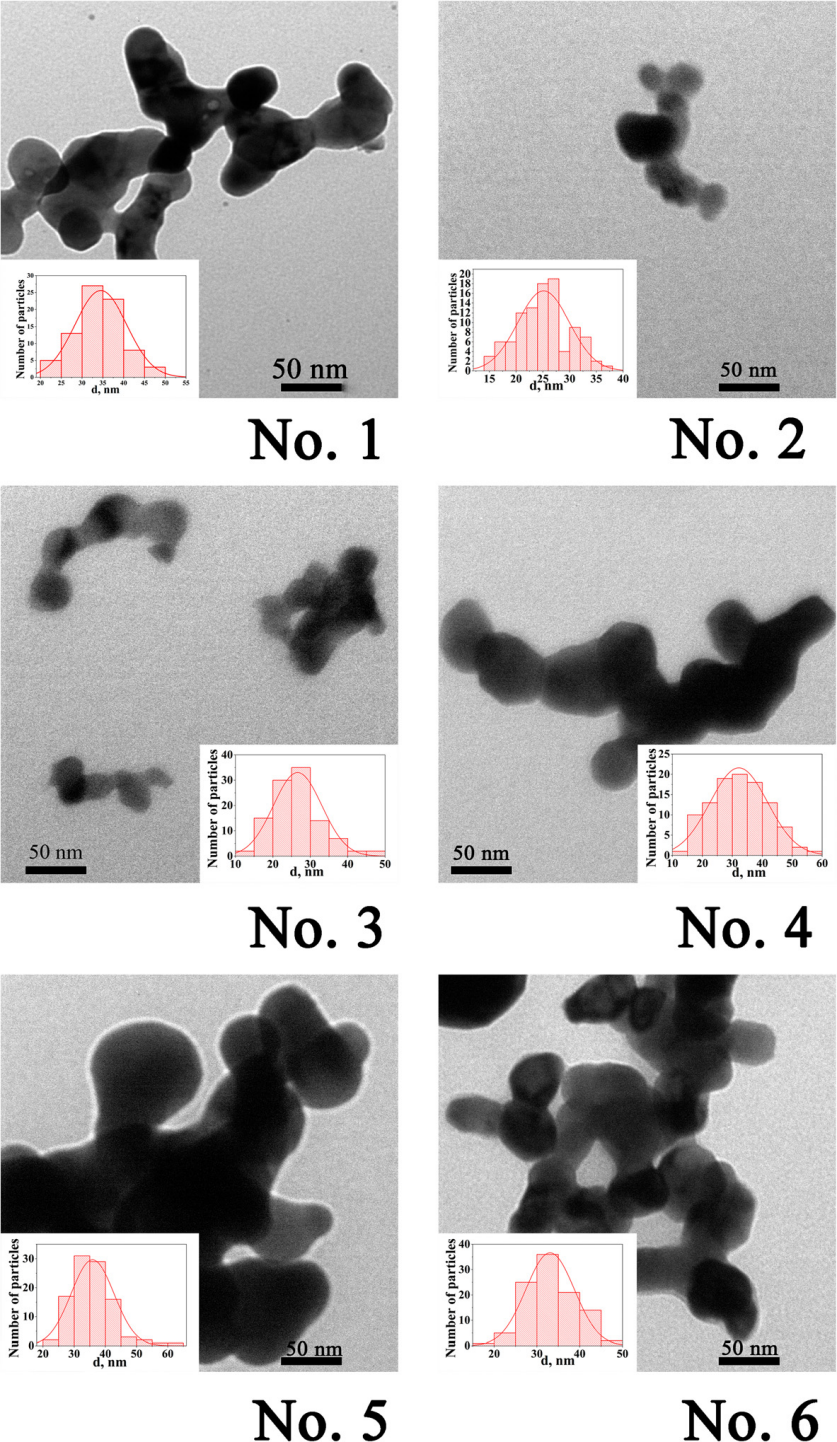
TEM images of La_0.77_Sr_0.23_Mn_1 *− y*_Fe_*y*_O_3_ nanoparticles: *No. 1 y* = 0; *No. 2 y* = 0.02; *No. 3 y* = 0.04; *No. 4 y* = 0.06; *No*. *5* *y* = 0.08; *No. 6 y* = 0.1

To investigate the morphology of nanoparticles, microstructural study was done by transmission electron microscope. Obtained results are shown in Fig. 3. An average particle size was determined, and particle size distribution was calculated as described in [21] (Table 2). An average size of La_0.77_Sr_0.23_Mn_1 *− y*_Fe_*y*_O_3_ (*y* = 0–0.1) nanoparticles lies in the range of 25–35 nm.

**Table 2:** Average size of La_0.77_Sr_0.23_Mn_1 − *y*_Fe_*y*_O_3_ (*y* = 0–0.1) nanoparticles synthesized by sol-gel method

Number	*y*	d. nm	σ. nm	σ. %
1	0	34	±6	18
2	0.02	25	±5	19
3	0.04	27	±6	24
4	0.06	32	±9	29
5	0.08	35	±6	19
6	0.10	33	±6	17

**Fig. 4:**
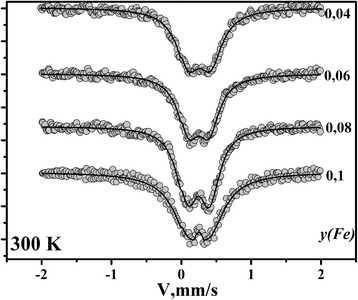
The Mössbauer spectra for La_0.77_Sr_0.23_Mn_1 *− y*_Fe_*y*_O_3_ nanoparticles

Mössbauer spectra for La_0.77_Sr_0.23_Mn_1 *− y*_Fe_*y*_O_3_ nanoparticles at 300 K are shown in Fig. 4. Based on the values of obtained isomer shift for all samples (~0.37 mm/s), it could be said that iron in these compounds is in the oxidation state 3+ [27].

It is known that magnetically ordered iron-containing materials display sextet in their Mössbauer spectra. For synthesized nanoparticles, the doublet formation is observed, independently on iron content. The reason for this may be either inhomogeneity of magnetic state or dynamic effects characteristic of superparamagnetic or paramagnetic states [10].

**Fig. 5:**
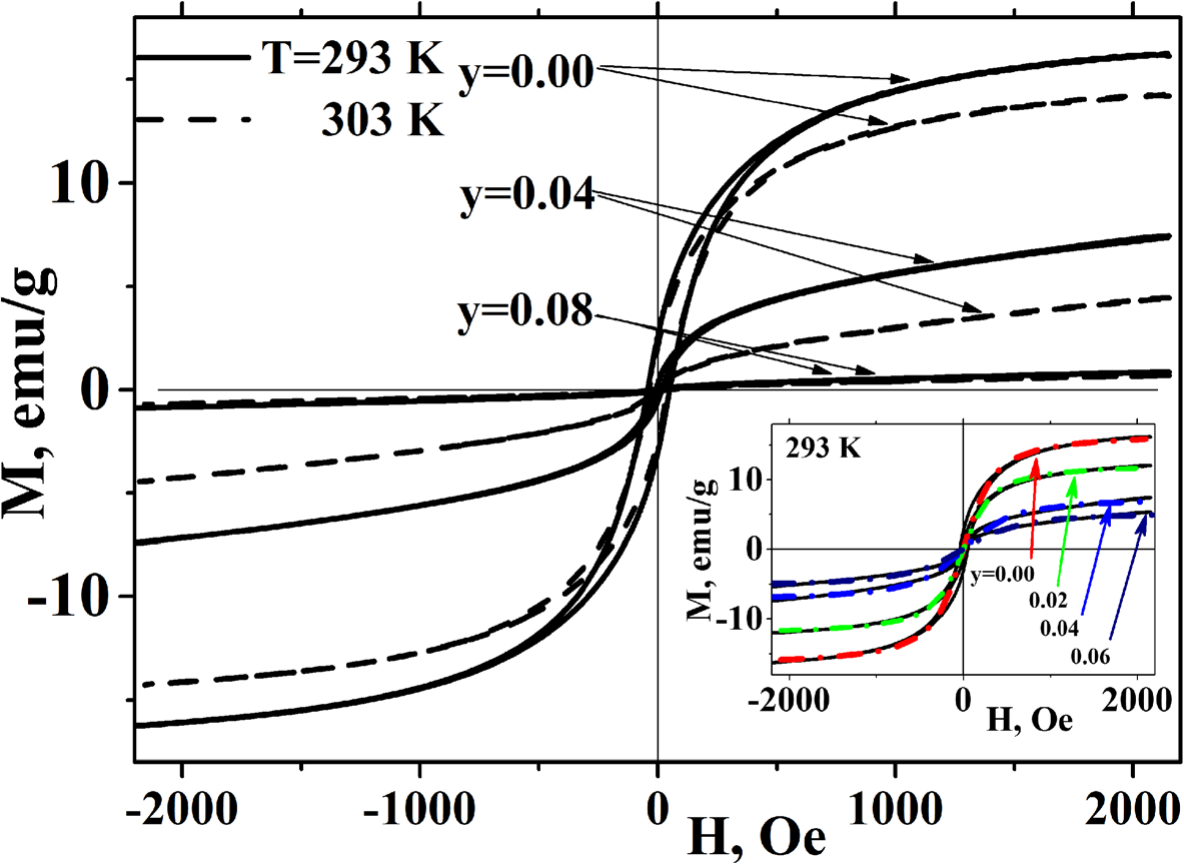
Magnetization of La_0.77_Sr_0.23_Mn_1 *− y*_Fe_*y*_O_3_ nanoparticles vs magnetic field at *T* = 293 and 303 K. Data for *y* = 0.02, 0.06, and 0.1 are omitted for clarity. An approximation of experimental data by Langevin function is shown in the *inset*

Field dependences of magnetization at *T* = 293 and 303 K are shown in Fig. 5. At 293 K, samples with higher iron content (*y* = 0.08–0.1) are characterized by small magnetization values (*M* < 1 emu/g) and almost linear *M*(*H*) dependence that is typical of paramagnetic state. The samples with *y* ≤ 0.06 have higher magnetization and tend to saturate in strong magnetic fields. Detailed analysis of the *M*(*H*) curves for samples with *y* = 0–0.06 shows that they can be well fitted by a Langevin function [28] (see inset to Fig. 5), which points towards the superparamagnetic behavior. At *T* = 303 K, only samples with *y* ≤ 0.02 magnetize superparamagnetically.

Figure 5 shows that at 293 and 303 K, a weak hysteresis with small coercivity and small residual magnetization (less than 20 % of the saturation magnetization) is observed for the samples with *y* ≤ 0.04. This can result from the size dispersion of nanoparticles or their partial agglomeration. Residual magnetization decreases with *y* increasing and becomes negligibly small for the samples with *y* ≥ 0.08.

Magnetic state of nanoparticles is usually quite inhomogeneous due to significant contribution of a surface layer whose properties differ from those of the volume properties and due to the scatter in particle sizes. It is shown in [29] that temperature behavior of nanoparticles ensemble can be satisfactory described by the introduction of an average Curie temperature *T*_*C*_ concept. It is expected in this case that the behavior of the magnetization of nanoparticles ensemble will obey the law $M(T)\sim \sqrt{T_C-T}$.

**Fig. 6:**
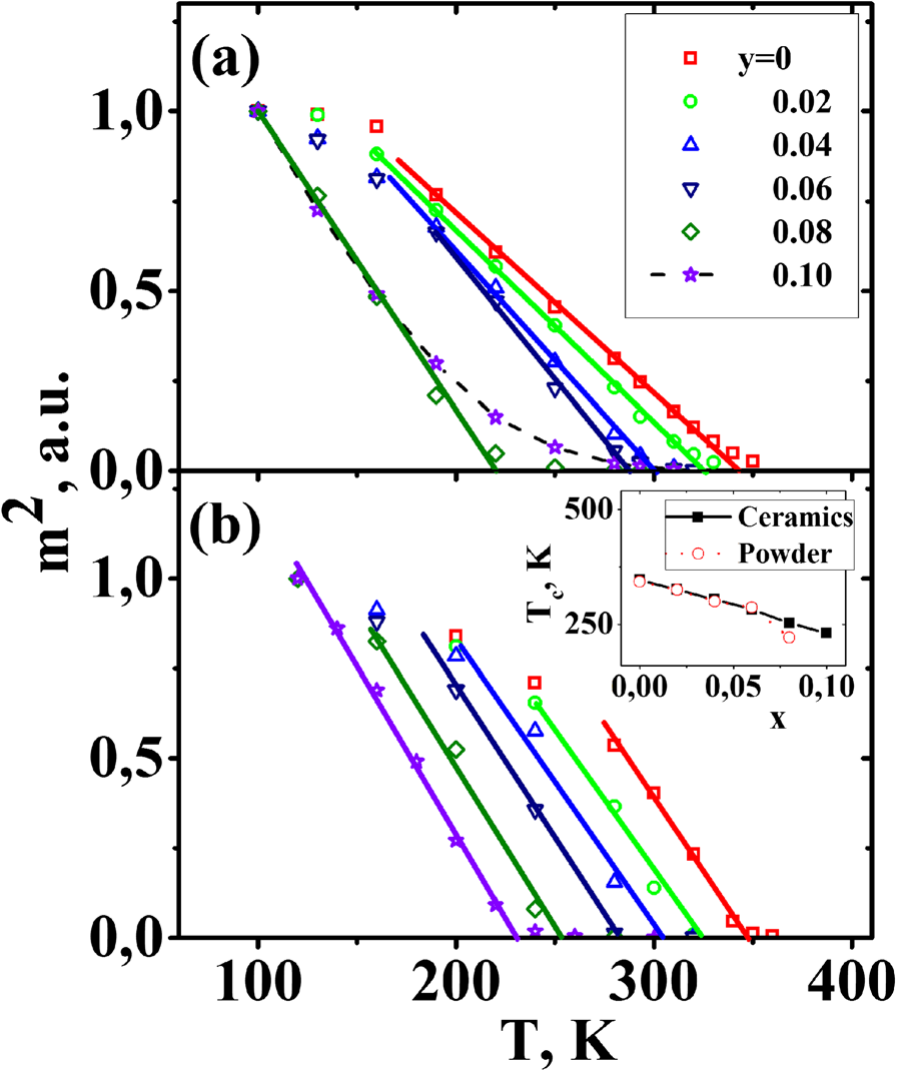
Temperature dependences of the square of normalized magnetization *m* = *M*(*T*, *H* = 6 kOe)/*M*(100 K, *H* = 6 kOe) for La_0.77_Sr_0.23_Mn_1 *− y*_Fe_*y*_O_3_ nanoparticles (**a**) and ceramic samples based on them (**b**). Concentration dependences of the Curie temperature for nanoparticles and ceramic samples are shown in the *inset*

Temperature dependences of the square of normalized magnetization *m* = *M*(*T*, *H* = 6 kOe)/*M*(100 K, *H* = 6 kOe) for La_0.77_Sr_0.23_Mn_1 *− y*_Fe_*y*_O_3_ nanoparticles are shown in Fig. 6a. It is observed that there is a wide temperature range where such dependence is linear. From these dependences (see Fig. 6a), it is possible to estimate the Curie temperature values by the points of intersection of linear area with the temperature axis. The highest Curie temperature is 343 K for the sample with *y* = 0. The Curie point for the samples with *y* ≤ 0.04 is higher than the room temperature, namely it is 325 and 300 K for the samples with *y* = 0.02 and 0.04, respectively. For the sample with *y* = 0.06, the Curie temperature is close to the room temperature and equals 287 K.

The Curie temperature for the sample with *y* = 0.08 is significantly lower than the room temperature (*T*_*C*_ = 221 K). At room temperature and higher temperatures, this sample is in the paramagnetic state even at strong magnetic fields. In the sample with *y* = 0.10, there is no region with the linear temperature dependence of the magnetization square. For this reason, it is impossible to correctly determine the Curie point for this sample. The magnetic state of this sample is strongly inhomogeneous.

To investigate the effect of the synthesis and heat treatment conditions on magnetic properties of Fe-doped manganites, the same investigations were performed for ceramic samples obtained from La_0.77_Sr_0.23_Mn_1 *− y*_Fe_*y*_O_3_ nanoparticles. Temperature dependences of the square of normalized magnetization *m* = *M*(*T*, *H* = 6 kOe)/*M*(100 K, *H* = 6 kOe) for La_0.77_Sr_0.23_Mn_1 *− y*_Fe_*y*_O_3_ ceramic samples are shown in Fig. 6b. The values of the Curie temperature were determined by means of the extrapolation of the linear parts of these dependences to the horizontal axis. The Curie points for the nanoparticles and corresponding ceramic samples are compared in the inset in Fig. 6b. It is seen that *T*_*C*_ values for corresponding samples are quite close, although in the case of nanoparticles, the magnetic transition is much wider (compare Fig. 6a and 6b).

Magnetization at room temperature and Curie temperature are important parameters which determine the heating efficiency of magnetics under the external alternative magnetic field. Table 3 collects these parameters for the nanoparticles and ceramics under investigation.

**Table 3:** Magnetic parameters of La_0.77_Sr_0.23_Mn_1 − *y*_Fe_*y*_O_3_ (*y* = 0–0.1) nanoparticles and ceramic samples based on them

*y*	*M*(*H* = 2 kOe; *T* = 293 K), emu/g		*T*_*C*_, K	
	Ceramic samples	Nanoparticles	Ceramic samples	Nanoparticles
0	51	16	347	343
0.02	39	12	326	325
0.04	13	7	304	300
0.06	3	2	283	287
0.08	1	0.7	253	221
0.10	0.8	0.8	231	−

**Fig. 7:**
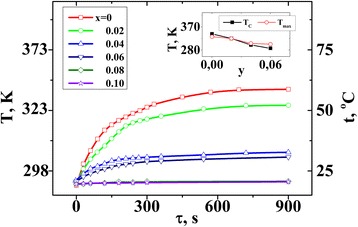
Temperature vs heating time dependences for magnetic fluids based on La_0.77_Sr_0.23_Mn_1 *− y*_Fe_*y*_O_3_ nanoparticles. The *inset* compares the Curie temperature *T*_*C*_ of the nanoparticles with the maximal heating temperature *T*_max_

Magnetic fluids based on synthesized La_0.77_Sr_0.23_Mn_1 *− y*_Fe_*y*_O_3_ (*y* = 0–0.1) nanoparticles and aqua solution of agarose were prepared according to [19]. It is established that the ability for manganites to heat up under an alternating magnetic field decreases with the increase of iron content in the sample (Fig. 7). The values of specific loss power calculated from the curves of Fig. 7 are summarized in Table 4.

**Table 4:** The specific loss power for La_0.77_Sr_0.23_Mn_1 − *y*_Fe_*y*_O_3_ (*y* = 0–0.1) nanoparticles under investigation

*y*	0	0.02	0.04	0.06	0.08	0.10
SLP. W/g	37	11	5	4	2	1

The results presented in Table 4 well correlate with the magnetic measurement data. At the initial stage of heating, the most effective heating is observed for the sample with *y* = 0, which has the greatest magnetizations, and least effective heating is characteristic of the samples with *y* = 0.08 and 0.10, which have the smallest magnetization (see Table 3).

It is important that after the abrupt initial temperature rise after turning on an alternating magnetic field, the curves in Fig. 7 reach saturation at a certain *T*_max_ value. The inset in Fig. 7 compares the values of *T*_max_ and Curie temperature *T*_*C*_ of the nanoparticles. It is observed that these values are quite close. However, it should be noted that *T*_max_ is not always exactly equal to *T*_*C*_, since *T*_max_ depends on both the features of heat exchange with environment and magnetic parameters of nanoparticles, in particular on the scatter in the values of magnetization and Curie temperature.

Based on the results of investigations, one can conclude that the action of an external magnetic field causes the effective heating of nanoparticles at temperatures lower than *T*_*C*_, while heating efficiency becomes strongly reduced upon a transition into the paramagnetic state. Thus, it is possible to control maximal temperature achieved during the heating by means of changing the Curie temperature of nanoparticles. This implies that the nanoparticles based on iron-doped manganites are promising as mediators of self-controlled magnetic nanohyperthermia.

## Conclusions

La_0.77_Sr_0.23_Mn_1 *− y*_Fe_*y*_O_3_ (*y* = 0, 0.02, 0.04, 0.06, 0.08, 0.1) nanoparticles were synthesized by sol-gel method. Ceramic samples based on these nanoparticles were obtained at sintering temperature 1613 K. XRD studies were carried out for all the samples, and crystallographic parameters (*a*, *c*, *V*) were calculated via Rietveld method. According to the XRD data, it is established that the unit cell volume for nanoparticles increases with the increase in iron content, which points towards the fact that heterovalent substitution of smaller Mn^4+^ ions by larger Fe^3+^ ions occurs. A reverse trend, observed for ceramic samples, implies that the isovalent substitution of larger Mn^3+^ ions by Fe^3+^ ions occurs.

According to Mössbauer spectroscopy results, it was established that iron in these compounds is in the oxidation state 3+. An average particle size calculated from microstructural data is in the range of 25–45 nm.

As a result of the studies of magnetic properties of La_0.77_Sr_0.23_Mn_1 *− y*_Fe_*y*_O_3_ samples, it was established that at room temperature, the nanoparticles and ceramic samples with *y* ≤ 0.06 exhibit superparamagnetic behavior and samples with *y* > 0.06 are paramagnetic. The values of Curie temperature *T*_*C*_ for nanoparticles and corresponding ceramic samples are very close. For both kinds of samples, the *T*_*C*_ values monotonously decrease with the increase in iron content, but magnetic transition is strongly broadened in nanoparticles compared to ceramic samples.

Magnetic fluids based on La_0.77_Sr_0.23_Mn_1 *− y*_Fe_*y*_O_3_ (*y* = 0, 0.02, 0.04, 0.06, 0.08, 0.10) nanoparticles and aqua solution of agarose were prepared. It is established that heating efficiency under an alternating magnetic field of nanoparticles becomes reduced as iron content grows.

It is shown that the action of an external magnetic field causes the effective heating of nanoparticles at temperatures lower than *T*_*C*_, while heating efficiency strongly weakens upon a transition into the paramagnetic state. Thus, fine tuning of the Curie temperature of nanoparticles allows control of the maximal temperature achieved during the heating.

Obtained results allow reliable prediction of magnetic parameters of La_0.77_Sr_0.23_Mn_1 *− y*_Fe_*y*_O_3_ system in the range of low *y* values (*y* = 0/0.1) that is very important for application of these materials in medicine, in particular as mediators of self-controlled magnetic nanohyperthermia.
